# Do antibacterial skin sutures reduce surgical site infections after elective open abdominal surgery? - Study protocol of a prospective, randomized controlled single center trial

**DOI:** 10.1186/s13063-019-3492-3

**Published:** 2019-07-02

**Authors:** Daniel Matz, Saskia Teuteberg, Andrea Wiencierz, Savas Deniz Soysal, Oleg Heizmann

**Affiliations:** 1 Department of General-, Visceral- and Thoracic Surgery, AGAPLESION Diakonie Hospital Rotenburg (Wuemme), Elise Averdieck-Str. 17, 27356 Rotenburg (Wuemme), Germany; 2 Department of Clinical Research, University of Basel, Clinical Trial Unit, University Hospital, Spitalstrasse 12, 4031 Basel, Switzerland; 3 Department of General Surgery, University of Basel Hospital, Spitalstrasse 21, 4031 Basel, Switzerland

**Keywords:** Surgical site infection, Antibacterial-coated suture material, Triclosan, Elective abdominal surgery, Skin closure

## Abstract

**Background:**

Surgical site infections (SSI) remain one of the most common complications in conventional abdominal surgery with an incidence between 4% and 19% (Sandini et al., Medicine (Baltimore) 95:e4057, 2016) in the literature. It is unclear whether the use of coated suture material for skin closure reduces the risk of SSI. In line with in-vitro results, we hypothesize that the use of antibacterial skin sutures (triclosan-coated poliglecaprone 25) reduces the rate of SSI after open abdominal surgery.

**Methods/design:**

To prevent SSI, triclosan-coated poliglecaprone 25 sutures will be tested against un-coated suture material for skin closure after elective open abdominal surgery of 364 patients. The study is planned as a single-center, prospective randomized controlled trial. Patients will be followed for 30 days after surgery to detect and document wound complications. The rate of SSI after 30 days will be analyzed in both groups.

**Discussion:**

If we can confirm the proposed hypothesis in our study, this could be a promising and feasible approach to lower SSI after open abdominal surgery. By lowering the rate of SSI this might offer a cost-saving and morbidity-reducing procedure.

**Trial registration:**

German Clinical Trials Register, DRKS00010047. Registered on 05.01.2017.

**Electronic supplementary material:**

The online version of this article (10.1186/s13063-019-3492-3) contains supplementary material, which is available to authorized users.

## Background

Surgical site infections (SSI) are a risk inherent to surgical procedures and a frequent complication, notably after digestive surgery. SSI are associated with severe morbidity and mortality, especially in high-risk patient populations [1, 2]. According to Center for Disease Control (CDC) guidelines [1], SSI up to 30 days after surgery are included, or even a year later when a foreign material such as a prosthesis has been implanted. SSI can be classified as superficial if they involve the skin only or subcutaneous tissue at the site of the incision, deep when they affect more internal structures of the abdominal layer, and organ-spaced when they also involve the abdominal cavity and the space between the organs that were manipulated or opened during the surgical procedure [3, 4]. The cornerstones for reducing the risk of SSI include exquisite surgical technique, timely and appropriate antimicrobial prophylaxis [5], effective and persistent skin antisepsis, and identification of adjunctive strategies for reducing wound contamination [6] and promoting wound healing. SSI are primarily caused by Gram-positive organisms from the patient’s own flora on the skin, mucous membranes, or hollow viscera manipulated during surgical procedures [1].

Various bacteria may contaminate not only the tissue in a surgical wound but also the actual suture material. If the surgical suture is implicated as the cause of a wound infection, then an antibacterial coating should nearly eliminate the possibility of the suture material becoming a vector of infection. The use of antibacterial-coated medical devices has become widespread. Numerous biomedical devices, including urologic and central venous catheters and orthopedic, vascular, and cardiothoracic implants, are commercially available with antibacterial impregnation or surface coatings.

It has been reported that percutaneous sutures approximating skin edges were often colonized from the body surface into the wound track by strains of *Staphylococcus epidermidis* capable of producing an amorphous extracellular matrix (biofilm), protecting the microbial populations from host defense factors [7]. Triclosan as a broad spectrum antiseptic is known from in vitro and in vivo studies to reduce the adherence of selective clinical Gram-positive, Gram-negative, drug-resistant, and biofilm-forming strains to the surface of a widely used braided surgical suture (polyglactin 910). Triclosan-coated polyglactin 910 sutures exhibit an antibacterial activity sufficient to prevent in vivo bacterial colonization in a guinea pig model [8]. Further, it is documented to be safe and efficient [2] and did not adversely affect wound healing in patients undergoing general surgical procedures [9]. In view of the high frequency of SSI and its substantial impact on morbidity and mortality, the reduction of the SSI rate should be a major priority among all surgical disciplines.

Therefore, our objective is to determine whether subcuticular closure of an abdominal wound with triclosan-coated Monocryl plus® running skin sutures influences the rate of SSI at 30 days following elective abdominal surgery when compared to similar uncoated Monocryl® sutures. Secondary objectives are to determine whether the use of triclosan-coated sutures reduce the length of hospital stay, wound dehiscence, and mortality rate at 30 days and number of re-operations in the same 30-day period following surgery.

In line with in vitro results, we hypothesize that the use of antibacterial skin sutures (with triclosan-coated poliglecaprone 25) reduces the rate of SSI after open elective abdominal surgery. The postulated mechanism states a reduced colonization of poliglecaprone 25 sutures by several strains of bacteria when antibacterial-coated suture material is used.

The study protocol has been written in accordance with the Standard Protocol Items: Recommendations for Interventional Trials (SPIRIT) checklist (Additional file 1).

## Methods/design

This single-center prospective randomized controlled trial (RCT) is conducted at the Department of General-, Visceral- and Thoracic Surgery and the Department of Obstetrics and Gynecology at the AGAPLESION Diakonie Hospital Rotenburg (Wuemme) gGmbH, Germany. It is designed as a double-blind parallel-group superiority trial.

**Fig. 1:**
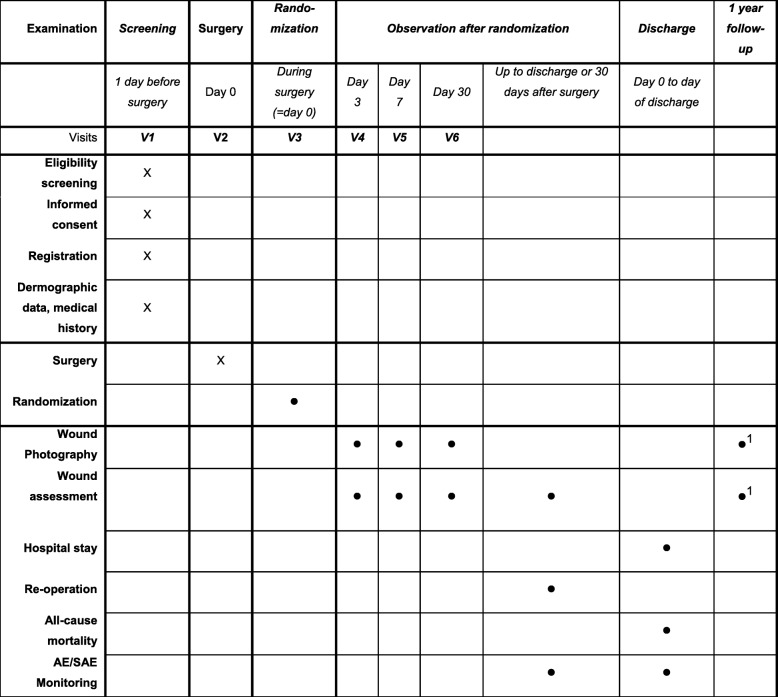
Schedule of assessments and procedures. Crosses indicate assessments for all registered patients and circles assessments of all randomized patients. ^1^Assessments for all registered patients with implanted foreign body during surgery

All patients planned for elective open general/colorectal or gynecological abdominal surgery will be consecutively informed about the study. Informed consent will be signed by each participating patient and a member of the study team at least 24 h before elective surgery. A flow diagram for this trial is shown in Fig. 1.

Baseline data such as gender, age, body mass index (BMI; calculated by Weight/Height^2^) will be collected from all included patients. Furthermore, factors that might influence the outcome of the surgery will be recorded. These factors include, but are not limited to, American Society of Anesthesiologists (ASA) classification, diabetes mellitus, antidiabetic medication, present immunosuppression, wound class according to CDC criteria (clean/clean-contaminated/contaminated/dirty), type of incision (transverse/longitudinal), operative time, amount of blood loss, length and orientation of incision.

### Randomization and intervention

After written informed consent is obtained, patients eligible for the study, will be randomized intraoperatively into two groups:

- Group A: Skin closure with non-coated suture material poliglecaprone 25 (Monocryl®, Ethicon GmbH, Norderstedt, Germany)
- Group B: Skin closure with triclosan-coated poliglecaprone 25 (Monocryl® Plus Ethicon GmbH, Norderstedt, Germany)

The randomization sequence is performed with sealed opaque envelopes. The patients are block randomized with equal block sizes of 20 items per block (allocation of patients per block is 1:1). The randomization list generation is computer-based using the *Random Allocations Software Version 1.0.0* (Freeware) by M. Saghaei, MD.

#### Surgical procedure

A perioperative antibiotic prophylaxis (standard dose) is administered to each patient 30–60 min prior to surgery (first or second class cephalosporin). Wound opening by the surgeon is performed in layers with:

- Skin incision with scalpel
- Preparation of the subcutaneous tissue with monopolar cautery, scalpel, or scissors
- Incision of the muscle fascia with diathermy, scalpel, or scissors

Wound closure is performed in layers with:

- Running sutures for the rectus fascia with Everett suture (Maxon® 1, Covidien IIc, Mansfield, USA)
- No subcutaneous sutures are added

A scrub nurse who is not involved in the patients’ follow-up will open the randomization envelope in the operation room and deliver the suture material to the scrub nurse immediately before wound closure. The scrub nurse hands over the sutures to the surgeon without showing the package of suture material. Macroscopically it is impossible to distinguish between Monocryl® and Monocryl® plus sutures. Consequently, the surgeon as well as the patients are unaware of the assigned treatment group.

Finally, intracutaneous skin closure with running stitches (using either Monocryl® or Monocryl® Plus) is performed by the surgeon and sterile wound dressing (Curapor, Lohmann & Rauscher, Germany) is applied.

In none of the cases will a wound drainage (superficial or deep) be set in place. According to the CDC guidelines [1], a follow-up of 30 days is required.

The operating surgeon, his team, and ward residents as well as the patients are unaware of assigned treatment groups. The operating surgeons and their teams will perform routine wound surveillance according to clinical standards, including diagnosis and treatment of SSI. Wound photographs will be taken on days 3 and 7 by the resident in charge, who is blinded as well.

The resident in charge on the ward is responsible for registration of SSI. If a SSI is detected by the resident, it needs to be verified by the consultant surgeon in charge. In addition, inpatients are seen regularly by members of the study team. Thus, appropriate sensitivity to detect in-hospital events can be provided. For post-discharge follow-up at day 30, well-trained investigators blinded to treatment allocation will examine any patient in an outpatient setting and document wound status by photograph. When SSI is present, clinically relevant microbiological samples are cultured as needed, and the patient receives standard wound therapy. In case of an implanted foreign body during surgery, an additional assessment including wound photography is performed in an outpatient setting one year after surgery.

To ensure appropriate specificity, all cases of SSI are validated by a board-certified wound care specialist who is blinded to the intervention.

### Outcome measures

#### Primary outcome measure

- Occurrence of any SSI within 30 days after surgery (within one year after implantation of foreign body)

In the present study, all types of SSI are considered for the primary outcome. SSI are defined as incisional (either superficial or deep) infection or organ-space infection according to CDC criteria [1]. Superficial incisional SSI include skin and subcutaneous tissues, deep incisional infections involve fascia and muscle, and organ-spaced infections involve any organ or space other than the incised layer of body wall that was opened or manipulated during surgery.

#### Secondary outcome measures

- Wound dehiscence and re-operation rate due to wound dehiscence within 30 days, all-cause 30-day mortality, and length of hospital stay
- Occurrence of any SSI within one year after implantation of a foreign body

Furthermore, we will evaluate potential risk factors for poor wound healing, such as gender, age, BMI, ASA classification, diabetes mellitus, antidiabetic medication, present immunosuppression, wound class according to CDC criteria, operative time, amount of blood loss, length, and orientation of incision [3, 10].

### Ethical considerations

The study was approved by the local ethics committee (Ethics Committee of the Georg-August University of Goettingen, Germany; 8/7/12). The study was registered at germanctr.de, ID DRKS00010047.

Informed consent, patients’ information, follow-up sheets, as well as the study protocol have been submitted.

All eligible patients will be precisely informed about the study and study-related procedures by a member of the study team. Patients declining participation in the trial will be excluded as the trial is strongly voluntary.

Both skin suture materials (Monocryl®, Monocryl® Plus) are officially available on the market in Germany and tested concerning safety [2]. Both are CE certified.

### Inclusion criteria

All patients from the Department of General-, Viceral- and Thoracic Surgery of AGAPLESION Diakonie Hospital Rotenburg (Wuemme), Germany and the Department of Gynecology and Obstetrics of the AGAPLESION Diakonie Hospital Rotenburg (Wuemme), Germany planned for open elective abdominal surgery ≥ 18 years old will be screened for inclusion.

Open abdominal surgery is defined as surgically opened peritoneal cavity.

### Exclusion criteria

- Participation in a clinical study in the last 3 months
- Factors limiting the ability to co-operate in the study
- Absence of signed informed consent before entering the study
- Any drug, alcohol, or nicotine abuse
- People with mental disorders
- Pregnant women
- Participants under 18 years
- Emergency surgery/procedures

### Statistics/sample size calculation

The sample size is estimated on the basis of a two-sided chi^2^ test for equality of the proportions of SSI occurrence within 30 days in the treatment and control groups (null hypothesis). Based on evidence in the literature, an infection rate of 12% is assumed for the control group and it is expected that the use of antibacterial sutures will reduce the SSI rate to 4% in the treatment group [11, 12].

Thus, a treatment effect of − 0.08 is assumed (i.e., an absolute risk reduction of 8 percentage points). Furthermore, the significance level of the test is set to 5% and a power of 80% is desired. To determine the sample size, we generate 10,000 synthetic data sets for a range of potential sample sizes using the above-mentioned assumptions for the SSI rates in both study groups. That is, we generate realizations of independent binomial random variables with probabilities of SSI occurrence p_T_ = 0.04 and p_C_ = 0.12, respectively. With each bi-variate data set, the two-sided chi^2^ test is performed at level α = 0.05.

Then, the power associated with a particular sample size n results in the H_0_-rejection rate across the 10,000 tests performed with data sets of size n. Hence, we obtain the power of the test as a function of the sample size and can identify the best value of n corresponding to a power of at least 80%. All computations are performed in the statistical software environment R (R Core Team, 2018, *R: A Language and Environment for Statistical Computing*. R Foundation for Statistical Computing, Vienna, Austria).

We find that, given a test level of α = 0.05, a total of 344 patients are needed to show that the SSI rates are different with a power of at least 80%. Furthermore, accounting for an expected drop-out rate of 5%, we obtain the number of *N* = 364 study participants to be recruited, that is, 182 patients for each study group.

All analyses will be performed after termination of the main part of the trial, that is, after the last 30-day follow-up visit has taken place.

The full analysis set (FAS) will include all study participants who were randomized and for whom data from at least one follow-up visit are available.

The per protocol set (PPS) will include all study participants in the FAS for whom the intervention was completed as planned and for whom complete data on all endpoints

are available.

All statistical analyses will be performed on the FAS according to the intention-to-treat principle. In addition, all analyses will be repeated using the PPS instead to assess the robustness of the findings.

The primary analysis investigates whether the SSI rate (within 30 days) among patients receiving antibacterial sutures is different from the rate in the control group, using a two-sided chi^2^ test for equality of proportions (null hypothesis) at significance level α = 0.05.

Besides the test result, the estimated SSI rates in the two groups and the estimated SSI rate difference will be reported with their 95% confidence intervals, the latter describing the precision of the estimates.

In addition to the primary analysis, we will assess the difference between the treatment and control groups when controlling for general risk factors for poor wound healing such as age and BMI of a patient or length and orientation of the incision. This will be achieved by evaluating the odds ratio obtained from estimating a multiple logistic regression model for the binary endpoint SSI (within 30 days) with explanatory factor treatment and adjusted for risk factors for poor wound healing. To further explore potential benefits of using antibacterial sutures, the differences between the treatment and control groups regarding the following secondary endpoints will be assessed:

- Wound dehiscence within 30 days (yes/no)
- Re-operation due to wound dehiscence within 30 days (yes/no)
- Death within 30 days (yes/no)
- Length of hospital stay (days)

The first three secondary outcomes will be analyzed estimating simple and multiple logistic regression models with explanatory factor treatment and, in the multiple case, adjusted for general risk factors for poor wound healing. The secondary endpoint length of hospital stay will be assessed using Poisson regression models instead.

For the subgroup of patients whose surgery involves the implantation of a foreign body, an additional long-term follow-up visit will take place one year after surgery. Once these data are collected, a final secondary analysis will be done for this subgroup of patients evaluating their SSI risk (within one year).

For all secondary analyses, parameter estimates and odds ratios will be reported together with 95% confidence intervals. If *p* values are reported, they will be interpreted as continuous measures of evidence against the associated null hypotheses.

### Study management

Data management is performed by ST and DM. All information required by the study protocol and collected during this trial will be entered in the electronic case report form (CRF; encrypted Excel database) and will be reviewed and signed by the investigator or subinvestigator. Quality control is going to be enforced by site visits and CRF review by the investigator or subinvestigator monthly. A close-out visit will be performed after enrollment of the very last patient.

The study steering committee comprises two senior consultant surgeons in general surgery. This is the main policy and decision-making committee for the study. The study is sponsored by the AGAPLESION Diakonieklinikum Rotenburg (Wuemme) and is therefore independent from commercial companies.

### Data management

The study is conducted according to the good clinical practice standards and legal regulations.

All data will be collected in an anonymous and encrypted database by the investigator at the AGAPLESION Diakonieklinikum Rotenburg/Wuemme, Germany. Data anonymization will be processed according to Keerie et al. [13]. The confidentiality of the participants will be maintained at any time. Therefore, CRFs must not contain personal data of the participating patients. Personnel from regulatory authorities and members of the ethics committees are firmly bound to respect confidentiality and to refrain from revealing the participants’ identity or any other personal information they might be aware of.

The investigator will maintain all study-related records (CRFs, medical records, informed consent documents, information regarding participants who discontinued, wound photographs, and other pertinent data). According to the applicable laws, data will be destroyed after a time period of 10 years after study termination.

The final trial data set will be securely transferred to the statistician at the Clinical Trial Unit at the University Hospital of Basel, Switzerland, who will be authorized for statistical analysis.

All individual participant data collected during the trial will be available from the corresponding author in an anonymized fashion on reasonable request after the study.

## Discussion

Surgical site infections account for 14 to 16% of all nosocomial infections in hospitalized patients [1]. Furthermore, as much as 5% of all patients undergoing surgical procedures develop SSI with the burden of additional mortality and morbidity despite different prevention schemes [14–16]. The first global guidelines for the prevention of surgical site infection were recently published by the WHO [17]. These guidelines address different measures to reduce SSI. The authors’ panel advocates the routine use of triclosan-coated sutures in surgical procedures independently of the type of surgery as a conditional recommendation, since there is a moderate quality of evidence in the analysed literature. The given recommendation is mainly based on the meta-analysis by Wu and colleagues [18]. It needs to be noted that the quality of the included RCTs was moderate or low and that some of the studies had an industrial sponsorship. Another weakness of this meta-analysis was the inclusion of different types of surgery (e.g., breast, vascular, orthopaedic, or colorectal surgery) which considerably differ in their SSI rates. Even emergency surgery was compared to elective surgery and open vs laparoscopic surgery, although one of the well documented benefits of laparoscopic surgery is the low rate of SSI [19] and an inverse relationship of SSI [20].

The meta-analysis by Henriksen [21] as well compared different types of surgery, such as elective open colorectal surgery, elective midline laparotomy, open appendectomy, and even laparotomies for fecal peritonitis. Edmiston et al. [22] and Chang et al. [23] pooled all available RCTs in their meta-analysis without stratifying the risk for wound class, type of operation, or organ/apparatus involved [11], as did Wu et al. [18] de Jonge et al. [24], Leaper et al. [25] as well as Hunger et al. [26].

Therefore, only patients undergoing open elective surgery were included in our trial. Most of the published RCTs and meta-analyses examined the effect on reduction of SSI of using antibacterial-coated suture material for fascial closure in abdominal surgery. Taking into account that the primary cause of SSI next to the bacterial flora of mucous membranes or hollow viscera is, in particular, the Gram-positive flora of the patient’s own skin [1], the focus of our research is on the choice of skin closure suture material to reduce SSI in abdominal surgery. Therefore, one of the potential strengths of our research is the use of antibacterial-coated suture material for the use of skin closure as triclosan is known to reduce the adherence of selective clinical Gram-positive, Gram-negative, drug-resistant, and biofilm-forming strains to the surface of the suture material. Moreover, triclosan is proven to be safe and does not affect wound healing [2].

To reduce postoperative SSI is undoubtedly of increasing interest as, apart from reduction of morbidity and mortality, hospitals are under pressure in terms of cost-effectiveness. SSI are further considered to reflect the quality of care in a hospital, as they are potentially preventable complications directly linked to surgery [10]. Therefore, it is of utmost interest to reduce postoperative SSI rates.

### Trial status

On 29th January 2016, final approval for the study was given by the local ethics committee. Consecutively, the first patient was enrolled on 12th February 2016. As of 12th April 2019, 280 patients were enrolled in the trial. We consider the intended number of patients will be reached in December 2019 at the latest.

## Additional file

Additional file 1: SPIRIT 2013 checklist: recommended items to address in a clinical trial protocol and related documents. SPIRIT Figure: recommended content for the schedule of enrolment, interventions, and assessments. (DOCX 30 kb)

## Data Availability

The datasets generated and/or analyzed during the current study will be available. from the corresponding author on reasonable request in an anonymized fashion after the end of the trial.

## Abbreviations

ASA: American Society of Anesthesiologists
CDC: Centers for Disease Control and Prevention
CE: Communauté Européenne
CRF: Case report form
CTU: Clinical trial unit
FAS: Full analysis set
PPS: Per protocol set
RCT: Randomized controlled trial
SSI: Surgical site infection

## References

[1] Mangram AJ, Horan TC, Pearson ML, Silver LC, Jarvis WR (1999). Guideline for prevention of surgical site infection, 1999. Hospital Infection Control Practices Advisory Committee. Infect Control Hosp Epidemiol.

[2] Jones RD, Jampani HB, Newman JL, Lee AS (2000). Triclosan: a review of effectiveness and safety in health care settings. Am J Infect Control.

[3] Malone DL, Genuit T, Tracy JK, Gannon C, Napolitano LM (2002). Surgical site infections: reanalysis of risk factors. J Surg Res.

[4] Horan TC, Gaynes RP, Martone WJ, Jarvis WR, Emori TG (1992). CDC definitions of nosocomial surgical site infections, 1992: a modification of CDC definitions of surgical wound infections. Infect Control Hosp Epidemiol.

[5] Weber WP, Marti WR, Zwahlen M, Misteli H, Rosenthal R, Reck S (2008). The timing of surgical antimicrobial prophylaxis. Ann Surg.

[6] Misteli H, Weber WP, Reck S, Rosenthal R, Zwahlen M, Fueglistaler P (2009). Surgical glove perforation and the risk of surgical site infection. Arch Surg.

[7] Gristina AG, Price JL, Hobgood CD, Webb LX, Costerton JW (1985). Bacterial colonization of percutaneous sutures. Surgery..

[8] Storch ML, Rothenburger SJ, Jacinto G (2004). Experimental efficacy study of coated VICRYL plus antibacterial suture in guinea pigs challenged with Staphylococcus aureus. Surg Infect.

[9] Ford HR, Jones P, Gaines B, Reblock K, Simpkins DL (2005). Intraoperative handling and wound healing: controlled clinical trial comparing coated VICRYL plus antibacterial suture (coated polyglactin 910 suture with triclosan) with coated VICRYL suture (coated polyglactin 910 suture). Surg Infect.

[10] Mujagic E, Zwimpfer T, Marti WR, Zwahlen M, Hoffmann H, Kindler C (2014). Evaluating the optimal timing of surgical antimicrobial prophylaxis: study protocol for a randomized controlled trial. Trials..

[11] Sandini M, Mattavelli I, Nespoli L, Uggeri F, Gianotti L (2016). Systematic review and meta-analysis of sutures coated with triclosan for the prevention of surgical site infection after elective colorectal surgery according to the PRISMA statement. Medicine (Baltimore).

[12] Justinger C, Moussavian MR, Schlueter C, Kopp B, Kollmar O, Schilling MK (2009). Antibacterial [corrected] coating of abdominal closure sutures and wound infection. Surgery..

[13] Keerie C, Tuck C, Milne G, Eldridge S, Wright N, Lewis SC (2018). Data sharing in clinical trials - practical guidance on anonymising trial datasets. Trials..

[14] Coello R, Charlett A, Wilson J, Ward V, Pearson A, Borriello P (2005). Adverse impact of surgical site infections in English hospitals. J Hosp Infect.

[15] Coskun D, Aytac J, Aydinli A, Bayer A (2005). Mortality rate, length of stay and extra cost of sternal surgical site infections following coronary artery bypass grafting in a private medical centre in Turkey. J Hosp Infect.

[16] Kirkland KB, Briggs JP, Trivette SL, Wilkinson WE, Sexton DJ (1999). The impact of surgical-site infections in the 1990s: attributable mortality, excess length of hospitalization, and extra costs. Infect Control Hosp Epidemiol.

[17] Leaper DJ, Edmiston CE (2017). World Health Organization: global guidelines for the prevention of surgical site infection. J Hosp Infect.

[18] Wu X, Kubilay NZ, Ren J, Allegranzi B, Bischoff P, Zayed B (2017). Antimicrobial-coated sutures to decrease surgical site infections: a systematic review and meta-analysis. Eur J Clin Microbiol Infect Dis.

[19] Lawson EH, Hall BL, Ko CY (2013). Risk factors for superficial vs deep/organ-space surgical site infections: implications for quality improvement initiatives. JAMA Surg.

[20] Hamza WS, Salama MF, Morsi SS, Abdo NM, Al-Fadhli MA (2018). Benchmarking for surgical site infections among gastrointestinal surgeries and related risk factors: multicenter study in Kuwait. Infect Drug Resist.

[21] Henriksen NA, Deerenberg EB, Venclauskas L, Fortelny RH, Garcia-Alamino JM, Miserez M (2017). Triclosan-coated sutures and surgical site infection in abdominal surgery: the TRISTAN review, meta-analysis and trial sequential analysis. Hernia..

[22] Edmiston CE, Daoud FC, Leaper D (2013). Is there an evidence-based argument for embracing an antimicrobial (triclosan)-coated suture technology to reduce the risk for surgical-site infections? A meta-analysis. Surgery..

[23] Chang WK, Srinivasa S, Morton R, Hill AG (2012). Triclosan-impregnated sutures to decrease surgical site infections: systematic review and meta-analysis of randomized trials. Ann Surg.

[24] de Jonge SW, Atema JJ, Solomkin JS, Boermeester MA (2017). Meta-analysis and trial sequential analysis of triclosan-coated sutures for the prevention of surgical-site infection. Br J Surg.

[25] Leaper DJ, Edmiston CE, Holy CE (2017). Meta-analysis of the potential economic impact following introduction of absorbable antimicrobial sutures. Br J Surg.

[26] Hunger R, Mantke A, Herrmann C, Mantke R. Triclosan-coated sutures in colorectal surgery: Assessment and meta-analysis of the recommendations of the WHO guideline. Chirurg. 2019;90(1):37–46. 10.1007/s00104-018-0732-0 30203169

